# Efficacy of avilamycin on the incidence and severity of diarrhea associated with pathogenic *Escherichia coli* and the subsequent growth performance of nursery pigs

**DOI:** 10.1093/tas/txae175

**Published:** 2024-12-14

**Authors:** Sarah A Weiland, Petra L Chang, Chris L Puls, Robert W Evelsizer, Scott N Carr, Brent Frederick, Sara Ebarb, Matt J Ritter

**Affiliations:** Elanco Animal Health, United States Farm Animal Team, Greenfield, IN 46140, USA; Elanco Animal Health, United States Farm Animal Team, Greenfield, IN 46140, USA; Elanco Animal Health, United States Farm Animal Team, Greenfield, IN 46140, USA; Elanco Animal Health, United States Farm Animal Team, Greenfield, IN 46140, USA; Elanco Animal Health, United States Farm Animal Team, Greenfield, IN 46140, USA; Cargill Animal Nutrition, North American Pork Team, Lewisburg, OH 45338, USA; Cargill Animal Nutrition, North American Pork Team, Lewisburg, OH 45338, USA; Cargill Animal Nutrition, North American Pork Team, Lewisburg, OH 45338, USA

**Keywords:** antibiotics, avilamycin, carbadox, nursery, pigs

## Abstract

A total of 3,329 commercial crossbred barrows and gilts were used to compare the efficacy of avilamycin on incidence and severity of diarrhea and growth performance of pigs naturally infected with *Escherichia coli*. An incomplete block design was used with a 2 × 4 factorial arrangement of treatments: 1) Stocking density (Single: 0.67 sq. m/pig vs. Double: 0.33 sq. m/pig) and 2) Nursery medication program [Nonmedicated control fed for 56 d (**CON**) vs. 80.5 mg/kg avilamycin fed for 21 d (**AVI21**) vs. 80.5 mg/kg avilamycin fed for 42 d (**AVI42**) vs. 55.1 mg/kg carbadox fed for 21 d (**CAR21**)]. Subjective diarrhea scores were collected weekly on each pen according to a 3-point scale. Pigs were weighed on days 0, 21, 42, and 56 of the study. Pigs had ad libitum access to feed and water throughout the study. Diets were formulated to meet or exceed NRC (2012) recommendations. Fecal swabs from pigs were collected and confirmed the presence of hemolytic *E. coli* within the first two weeks of study. During the first 21 d, feeding AVI21 and AVI42 lowered (*P* < 0.05) diarrhea severity compared to controls, with CAR21 being intermediate. In the second 21 d, feeding AVI42 lowered (*P* < 0.05) diarrhea incidence and severity compared to the other treatments. In general, feeding medication resulted in lower maximum diarrhea scores compared to nonmedicated controls. For the overall study period (day 0 to 56), there were no (*P* > 0.05) stocking density × nursery medication program interactions for growth performance measures. A significant interaction (*P* < 0.05) was observed for day 0 to 42; feed conversion did not differ between double and single stocking density treatments for AVI42, whereas for CON, AVI21, and CAR21, feed conversion was lower in the single stocking density treatment. For the overall study period, there was no effect (*P* > 0.05) of nursery medication program on final BW, ADG, ADFI, or incidence of morbidity and mortality. Overall G:F was improved (*P* < 0.05) for pigs fed avilamycin compared to carbadox, with controls being intermediate. Double stocking resulted in lower overall ADFI (*P* < 0.05) and improved G:F (*P* < 0.05) compared to single stocking, but increased overall morbidity and mortality and diarrhea severity, incidence, and overall scores (*P* < 0.05). The results of this study demonstrate relatively similar performance with improved feed conversion and improvements in diarrhea incidence and severity for pigs fed avilamycin compared to carbadox.

## Teaser

This study demonstrates similar performance with improved feed conversion when feeding avilamycin to pigs naturally infected with *Escherichia coli* , regardless of stocking density, and emphasizes the impact of customizing medication to match the severity and duration of the disease challenge.

## Introduction

*Escherichia coli* (*E. coli*) continues to present challenges in the swine industry, particularly in nursery pigs, where it impacts not only the animal health and well-being but ultimately the productivity and profitability of swine operations. While some strains of *E. coli* bacteria are harmless, certain strains can cause a variety of diseases, including postweaning diarrhea and edema disease. Due to the cost associated with treatment, slower growth, and mortality, diseases from *E. coli* remain economically important to the swine industry and thus, an area of focus for intervention research.

Globally, common feed programs such as pharmacological levels of zinc oxide and various medications have been restricted or eliminated as available tools to producers. In addition, regulatory pressures over some proven antibiotics, like carbadox, have led to further evaluation of alternative antibiotics. Avilamycin was approved by the FDA in 2015 for use in U.S. swine diets for the reduction in incidence and overall severity of diarrhea in the presence of pathogenic *E. coli*, and previous work has shown avilamycin and carbadox have similar impacts on pig performance when fed in the presence of *E. coli* (Carvajal et al., 2014; FDA FOI, 2015; Greiner and Knopf, 2018). However, relatively little controlled commercial research has been conducted evaluating avilamycin in the United States since its approval.

Furthermore, different housing and management programs used by U.S. swine producers during the nursery period may exacerbate disease pressure. A relatively common practice in the U.S. swine industry, particularly in wean-to-finish commercial facilities, is to overstock pigs at weaning, then subsequently move a portion of those pigs to another finishing facility as pigs grow larger. The practice, known as “double stocking,” has been shown to temporarily reduce floor space and feeder space per pig, resulting in some reduction in feed intake and gain, to increase maximum facility throughput (e.g., kg of pork produced per square meter of facility; Wolter et al., 2002). However, previous work by Li et al. (2020) suggests increasing stocking density can lead to altered immune system function, which may impact the severity of disease and/or the efficacy of interventions. Therefore, the objective of this study was to compare the impact of carbadox fed for 21 d (which was current practice at that site at the time of the trial) to avilamycin fed by general label instruction of 21 d, and an extended 42-d treatment of avilamycin under veterinary feed directive in a natural *E. coli* challenge and understand if stocking density has any impact on diarrhea incidence, performance, and medication intervention efficacy.

## Materials and Methods

Experimental procedures were approved by the Elanco Institutional Animal Care and Use Committee prior to the start of the study (IACUC #1520). Avilamycin (Kavault, Elanco Animal Health, Indianapolis, IN) is indicated for reduction in incidence and overall severity of diarrhea in the presence of pathogenic *E. coli* in groups of weaned pigs and was fed according to label instructions at 80.5 mg/kg of feed for up to 42 consecutive days based on veterinary directive. Carbadox (Mecadox, Phibro Animal Health, Teaneck, NJ) is indicated for the control of swine dysentery and bacterial swine enteritis and was fed according to label instructions at 55.1 mg/kg for 21 d, which was the standard medication program used for enteric health and performance on the farm at that time.

### Experimental Design and Treatments

The study was carried out for a fixed time of 56 d from 6.7 ± 0.57 kg to 25.5 ± 2.23 kg of BW using 3,329 commercial crossbred pigs and an incomplete block design (blocking factors were initial BW, room, and location within room) with a 2 × 4 factorial arrangement of treatments: 1) Stocking density (Single: 32 pigs per pen; 0.67 sq. m/pig vs. Double: 64 pigs per pen; 0.33 sq. m/pig) and 2) Nursery medication program [Nonmedicated control fed for 56 d (**CON**) vs. 80.5 mg/kg avilamycin fed for the first 21 d (**AVI21**) vs. 80.5 mg/kg avilamycin fed for the first 42 d (**AVI42**) vs. 55.1 mg/kg carbadox fed for the first 21 d (**CAR21**)]. Once dietary treatments were complete, all pigs were fed the common control diet. The study was conducted in a standard commercial wean-to-finish research facility with 2 rooms, each consisting of 36 pens available for use on the study. Within each room, there were 4 complete replicates of each of the 2 × 4 treatment interaction subclasses and, due to space constraints in the facility, 1 additional replicate of each medication program treatment housed at 32 pigs per pen. Therefore, there were 10 pens of each medication program treatment housed at the single stocking density and 8 pens of each medication program treatment housed at the double stocking density. Pen was the experimental unit for all diarrhea and growth performance measurements.

Antibiotics were fed according to the veterinary feed directive and in accordance with their FDA-approved product labels, including labeled dosage and durations.

### Animals and Allotment to Study

A total of 3,329 crossbred barrows and gilts that were the progeny of DNA 600 sires × DNA 241 dams (DNA Genetics, Columbus, NE) were used in the study. There were two rooms at the research facility. Each room was filled with approximately 1,700 pigs one week apart.

Allotment to the study was carried out at weaning (approximately 21 d of age). Upon arrival at the facility, pigs were sorted by weight (visual appraisal) into heavy, medium, and light categories. Within each room and after sorting, 20 pens of 32 pigs (single stocking density) and 16 pens of 64 pigs (double stocking density) were randomly formed with equal numbers of heavy, medium, or light pigs. After pen conformation was complete, all pens were weighed as a group. The heaviest 4 pens of 32 pigs and heaviest 4 pens of 64 pigs were assigned to replicate 1, the next 4 pens of each assigned to replicate 2, and so on until there were 4 complete replicates within each room of the 2 × 4 treatment interaction subclasses. The remaining lightest 4 pens of 32 pigs were assigned to replicate 5. Pens within a replicate were moved to adjacent pens within the facility. Within replicate and stocking density treatment, pens were randomly allotted to one of the 4 medication program treatments and immediately began the study.

### Housing and Management

Pigs were housed in a tunnel-ventilated, curtain-sided facility with fully slatted concrete floors. Pen dimensions provided a usable floor space of 21.3 m^2^ per pen. Each pen was equipped with a 4-space single-sided dry box feeder mounted on the pen division that provided 122 cm of total linear feeder space and two cup water drinkers.

### Diets, Feeding, and Treatments

A 4-phase dietary nursery program was used during the study: Phase 1: fed 1.81 kg/pig, Phase 2: fed 5.44 lb/pig, Phase 3: fed to day 42 of study, and Phase 4: common phase fed to day 56 of study. Nursery medications were added to the diets at the expense of corn. Diet formulations and calculated composition of the diets fed during the experimental period are presented in Table 1. Diets were assayed to confirm medication levels in feed. Pigs had ad libitum access to feed and water throughout the study period.

**Table 1.** Experimental diet formulation by phase, as-fed basis

	Phase 1			Phase 2			Phase 3		Phase 4
Ingredient, %	Control	CAR^1^	AVI^2^	Control	CAR	AVI	Control	AVI	Control
Corn	30.5	29.6	30.5	44.4	43.5	44.4	56.6	56.6	46.9
Soybean meal	22.3	22.4	22.3	30.9	31.0	30.9	27.4	27.4	27.7
Corn DDGS	–	–	–	3.5	3.5	3.5	10.0	10.0	10.0
Bakery meal	–	–	–	–	–	–	–	–	10.0
Oats	15.0	15.0	15.0	2.5	2.5	2.5	–	–	–
Whey ingredients	13.4	13.4	13.4	6.7	6.7	6.7	–	–	–
Fat	3.4	3.4	3.4	2.5	2.5	2.5	2.5	2.5	2.7
Processed soy	1.8	1.8	1.8	2.0	2.0	2.0			
Soybean hulls	1.5	1.5	1.5	1.0	1.0	1.0			
Plasma	2.0	2.0	2.0	–	–	–			
Crystalline amino acids	1.4	1.4	1.4	1.0	1.0	1.0	1.0	1.0	0.7
Monodicalcium phosphate	1.1	1.1	1.1	1.3	1.3	1.3	0.6	0.6	0.5
Calcium carbonate	0.8	0.6	0.8	1.0	0.8	1.0	1.0	1.0	1.0
Salt	0.5	0.5	0.5	0.4	0.4	0.4	0.6	0.6	0.4
Zinc oxide	0.4	0.4	0.4	0.4	0.4	0.4			
Phase 1, other^3^	5.9	5.9	5.9						
Phase 2, other^3^				2.5	2.5	2.5			
Phase 3, other^4^							0.3	0.3	
Phase 4, other^4^									0.2
Kavault (avilamycin 20%)	–	–	0.04	–	–	0.04	–	0.04	–
Mecadox 2.5 g/lb	–	1.0	–	–	1.0	–	–	–	–
Total	100	100	100	100	100	100	100	100	100
Calculated composition									
Crude protein, %	19.8	19.8	19.8	21.4	21.4	21.4	20.6	20.6	20.9
Fat, %	6.7	6.7	6.7	5.0	5.0	5.0	5.5	5.5	6.6
Lysine, %	1.51	1.51	1.51	1.50	1.50	1.50	1.48	1.48	1.42
Fiber, %	2.26	2.48	2.26	2.49	2.71	2.49	2.63	2.63	2.64
Ca, %	0.76	0.76	0.76	0.78	0.78	0.78	0.60	0.60	0.58
P, %	0.66	0.66	0.66	0.67	0.67	0.67	0.51	0.51	0.50
Na, %	0.40	0.40	0.40	0.27	0.27	0.27	0.25	0.25	0.22
Cu, mg/kg	126	126	126	126	126	126	125	125	152
Zn, mg/kg	3,026	3,026	3,026	2,990	2,990	2,990	100	100	84
Net energy, kcal/kg	2,496	2,476	2,495	2,389	2,369	2,388	2,399	2,398	2,476

^1^CAR = dietary treatment containing carbadox (Mecadox, Phibro Animal Health, Teaneck, NJ).

^2^AVI = dietary treatment containing avilamycin (Kavault, Elanco Animal Health, Indianapolis, IN).

^3^Other = Nursery concentrate ingredients (organic acids, antioxidants, flavors, phytase, and other feed additives); vitamins and trace minerals at levels meeting or exceeding NRC (2012) recommendations.

^4^Other = Vitamins and trace minerals at levels meeting or exceeding NRC (2012) recommendations and phytase.

The presence of *E. coli* was confirmed by fecal swabs collected from pigs showing clinical signs of enteric disease, and diarrhea incidence scores confirmed pigs were under gastrointestinal stress during the study. Weekly diarrhea scores were collected on days 8, 15, 22, 29, 36, 43, 50, and 57 (day after final weight collection) of study. Each pen was assigned a scour score using a 3-point scale: 0 = < 5% of pigs or feces with signs of scours; 1 = 5% to 25% of pigs or feces with signs of scours; 2 = greater than 25% of pigs or feces with signs of scours. Incidence of diarrhea was calculated as pens with scores of 1 or 2 at any point during the study. The evaluator was not blinded to medication treatments. Due to high diarrhea scores of pigs on the control treatment, the decision was made by the attending veterinarian to treat all animals with neomycin in the water starting on day 25 of the study for 5 consecutive days.

### Growth Study Measurements

All pigs were weighed as a group (i.e., pen) on days 0, 21, 42, and 56 of study. All feed additions to the feeders were recorded and the amount of feed remaining in the feeders was weighed at the time of pig weighing and used to calculate ADFI and G:F.

Incidence of morbidity and mortality was recorded for all pigs throughout the study. Morbidity was defined as any pig that was removed from the study for any reason other than mortality. Mortality was defined as any pig that died while on study. Research technicians responsible for removing animals from the study were not blinded to experimental treatments. All mortalities and morbidities were weighed at the time of removal from study, and weights were used to calculate total pig days for each pen in growth performance calculations.

### Statistical Analysis

The PROC UNIVARIATE procedure of SAS (SAS Inst. Inc., Cary, NC) was used to verify normality and homogeneity of variance of the variables. All growth performance variables conformed to normality assumptions and were analyzed using PROC MIXED of SAS. The pen of pigs was the experimental unit for growth performance measurements (BW, ADG, ADFI, G:F). The Kenward–Rogers adjustment was made to account for the differences in degrees of freedom between the single stock (*n* = 40) and double stock (*n* = 32) treatments. The model included the fixed effects of stocking density, nursery medication program, and the two-way interaction, and the random effect of room and replicate within room. LSMEANS were differentiated using the pdiff option of SAS. Significance between treatments was defined as *P* ≤ 0.05, while 0.05 <* P *≤ 0.10 was considered a trend.

For the incidence of morbidity and mortality and diarrhea severity, incidence, and mean score, which did not meet normality assumptions, data were summarized by pen and analyzed using a generalized linear mixed model and the PROC GLIMMIX procedure of SAS. The Kenwood–Rogers adjustment was made to account for the differences in degrees of freedom between the single-stock and double-stock treatments. The model included the fixed effects of stocking density, nursery medication treatment, the two-way interaction, and the random effect of room and replicate within room. LSMEANS were differentiated using the pdiff option of SAS. Significance between treatments was defined as *P* ≤ 0.05, while 0.05 <* P *≤ 0.10 was considered a trend.

## Results and Discussion

### Impact of Medication Program and Stocking Density on Diarrhea Score and Incidence

From day 0 to 21, a medication program × stocking density interaction was observed for maximum diarrhea severity score. Maximum diarrhea severity scores were greater (suggesting more severe diarrhea) in double-stocked pens fed CON, AVI21, and CAR21 medication programs compared to single-stocked pigs on the same programs. However, double-stocked pigs fed AVI42 had similar maximum diarrhea scores to single-stocked pigs, regardless of medication program (*P* < 0.05, Table 2). A similar pattern was observed for mean diarrhea score, as double-stocked pens fed AVI21 or AVI42 had lower mean diarrhea scores than double stock pigs on the CAR21 and control programs, with no differences observed between medication programs when pigs were single stocked (*P* < 0.05, Table 2). This suggests that double stocking may exacerbate early nursery enteric disease in pigs, and feed medication programs may offer differing levels of effectiveness at mitigating clinical signs of disease.

**Table 2.** Effects of nursery medication program and stocking density on incidence and severity of diarrhea in nursery pigs

	Medication program^1^					Stocking density			*P*-value		
Item	Control	AVI21	AVI42	CAR21	SEM^2^	Double	Single	SEM	Med	Density	Med × Density
No. pens	18	18	18	18	-	32	40	–	–	–	–
Diarrhea scores^3^											
Days 0 to 21											
Max score^4^	0.88^a^	0.36^b^	0.44^b^	0.59^ab^	0.111	0.78^a^	0.35^b^	0.157	0.01	<0.001	0.04
Double	1.25^a^	0.63^bc^	0.38^cd^	0.88^ab^	0.157	–	–	–	–	–	–
Single	0.50^bcd^	0.10^d^	0.50^bcd^	0.30^cd^	–	–	–	–	–	–	–
Incidence^5^	75.0	36.3	43.8	58.8	10.53	71.9^a^	35.0^b^	7.44	0.06	0.001	0.07
Mean diarrhea score^6^	0.39^a^	0.14^c^	0.18^bc^	0.33^ab^	0.060	0.35^a^	0.17^b^	0.042	0.01	0.003	0.04
Double	0.58^a^	0.25^bc^	0.12^c^	0.46^ab^	0.084	–	–	–	–	–	–
Single	0.20^c^	0.03^c^	0.23^bc^	0.20^c^	–	–	–	–	–	–	–
Days 21 to 42											
Max score^4^	0.91^a^	0.70^a^	0.24^b^	0.85^a^	0.136	0.88^a^	0.48^b^	0.099	0.002	0.004	0.81
Incidence^5^	75.0^a^	57.5^a^	23.8^b^	78.8^a^	10.33	75.0^a^	42.5^b^	7.30	0.001	0.003	0.73
Mean diarrhea score^6^	0.44^a^	0.40^a^	0.07^b^	0.41^a^	0.069	0.49^a^	0.17^b^	0.050	<0.001	<0.001	0.07
Days 0 to 42											
Max score^4^	1.14^a^	0.75^bc^	0.56^c^	0.96^ab^	0.134	1.03^a^	0.68^b^	0.095	0.02	0.01	0.74
Incidence^5^	85.0	62.5	56.3	90.0	10.30	84.4^a^	62.5^b^	7.27	0.06	0.04	0.94
Mean diarrhea score^6^	0.43^a^	0.28^ab^	0.12^b^	0.37^a^	0.053	0.43	0.17	0.038	0.001	<0.001	0.02
Double	0.62^a^	0.48^a^	0.11^b^	0.50^a^	0.074	–	–	–	–	–	–
Single	0.23^b^	0.08^b^	0.13^b^	0.24^b^	–	–	–	–	–	–	–
Days 0 to 56											
Max score^4^	1.14^a^	0.81^ab^	0.62^b^	0.96^ab^	0.130	1.09^a^	0.68^b^	0.094	0.04	0.002	0.73
Incidence^5^	85.0	68.8	62.5	90.0	10.19	90.6^a^	62.5^b^	7.43	0.17	0.01	0.93
Mean diarrhea score^6^	0.36^a^	0.25^a^	0.10^b^	0.30^a^	0.047	0.37^a^	0.14^b^	0.035	0.001	<0.001	0.01
Double	0.53^a^	0.44^a^	0.10^b^	0.41^a^	0.065	–	–	–	–	–	–
Single	0.19^b^	0.06^b^	0.11^b^	0.18^b^	–	–	–	–	–	–	–

^1^Medication: Control = no nursery medication; AVI21 = Avilamycin (Kavault) fed at 80.5 mg/kg for 21 d; AVI42 = Avilamycin (Kavault) fed at 80.5 mg/kg for 42 d; CAR21 = Carbadox (Mecadox) fed at 55.1 mg/kg for 21 d.

^2^SEM listed for significant medication program × stocking density interactions are pooled SEM.

^3^Diarrhea scores recorded on a 3-point scale: 0 = no diarrhea; 1 = 5-25% of feces show signs of diarrhea; 2 = > 25% of feces show signs of diarrhea.

^4^Max score = The max score (0, 1, or 2) was determined from all sampling points. The single max score was calculated for all pens, and the number shown is the LSMEAN of all max scores.

^5^Incidence was determined as Yes/No, where Yes = a score of 1 or 2 at any point during the study period. The LSMEAN presented is the percentage of pens showing incidence of diarrhea during the study.

^6^Mean diarrhea score was calculated as the mean of weekly sampling time points. The single mean score was calculated for all pens, and the number shown is the LSMEAN of all mean diarrhea scores.

^abcd^Means within a row with different superscripts are different (*P* < 0.05).

From days 21 to 42, diarrhea max score, incidence, and mean diarrhea score were all lowest in the AVI42 treatment (*P* < 0.05; Table 2). This is not surprising as AVI42 was the only program with medication still in diets from days 21 to 42. These results suggest when enteric disease pressure continues past 21 d in the nursery, continued feed medication may be beneficial in supporting pigs through those challenges. Neomycin was also supplemented during this time period to support pigs, specifically in the control group, through the *E. coli* challenge. Neomycin is recommended for treatment and control of colibacillosis caused by *E. coli* in pigs. As that treatment was applied to all pigs, regardless of medication program, the trial results are not confounded by the supplementation of neomycin.

Over the entire treatment period (dyas 0 to 42), the AVI42 medication program had the lowest maximum diarrhea severity score, followed by the AVI21 and CAR21 programs, then controls (*P* < 0.05; Table 2). For the overall trial period (days 0 to 56), an interaction similar to that previously discussed was observed for mean diarrhea score in which double-stocked pigs fed the AVI42 program had significantly lower mean diarrhea scores than double-stocked pigs fed the other medication programs, while there was no impact of medication program on diarrhea scores in single stocked pigs (*P* < 0.05; Table 2). For the entire trial period, double-stocked pigs reported higher maximum diarrhea scores and greater diarrhea incidence than single-stocked pigs, regardless of medication program (*P* < 0.05; Table 2). These results support previous work indicating that increased stocking density may increase animal stress levels and disease pressure, thus changing how the animal responds to disease (Li et al., 2020). While we did not evaluate stress or disease pressure in this study, diarrhea scores would indicate that double-stocked pigs experienced an exacerbated challenge state. Feeding avilamycin for 42 d appeared to lessen the challenge state experienced by the pigs, as indicated by lower diarrhea scores similar to levels of their single-stocked counterparts.

### Effects of Nursery Medication Program, Stocking Density, and Their Interaction on Growth Performance

Pigs fed AVI42 and CAR21 programs had higher ADG and tended to have higher BW at day 21 compared to the control program, with AVI21 being intermediate (*P* < 0.05; Table 3). Feed efficiency was improved in all medication programs compared to controls from days 0 to 21, and double-stocked pigs had greater G:F than single-stocked pigs (*P* < 0.05; Table 3). There was no impact of medication program or stocking density on ADFI during this period.

**Table 3.** Effects of nursery medication program and stocking density on growth performance

	Medication program^1^				SEM^2^	Stocking density		SEM	*P*-value		
Item	Control	AVI21	AVI42	CAR21		Double	Single		Med	Density	Med × Density
No. pens	18	18	18	18	-	32	40	–	–	–	–
Start BW, kg	5.5	5.5	5.5	5.5	0.10	5.5	5.5	0.10	1.00	0.70	0.84
Days 0 to 21											
Day 21 BW, kg	11.1	11.5	11.7	11.7	0.26	11.7	11.4	0.22	0.10	0.12	0.74
ADG, g/d	263.1^b^	281.2^ab^	294.8^a^	290.3^a^	9.1	290.3	276.7	7.3	0.04	0.12	0.73
ADFI, g/d	353.8	353.8	362.9	371.9	10.9	353.8	367.4	9.1	0.50	0.12	0.82
G:F	0.746^b^	0.794^a^	0.808^a^	0.787^a^	0.013	0.813^a^	0.754^b^	0.010	0.004	<0.001	0.26
Days 21 to 42											
Day 42 BW, kg	23.8	24.1	24.7	24.4	0.51	24.4	24.1	0.44	0.32	0.53	0.54
ADG, g/d	598.7	598.7	612.3	598.7	12.2	598.7	603.3	10.9	0.64	0.60	0.36
ADFI, g/d	861.8	861.8	870.9	875.4	17.2	852.8	880.0	15.0	0.85	0.06	0.75
G:F	0.695^ab^	0.696^ab^	0.703^a^	0.685^b^	0.004	0.703^a^	0.687^b^	0.003	0.04	<0.001	0.16
Days 42 to 56											
Day 56 BW, kg	34.5	34.9	35.8	35.2	0.65	34.8	35.4	0.59	0.15	0.20	0.63
ADG, g/d	789.3	798.3	821.0	802.9	12.2	775.6^b^	830.1^a^	10.9	0.06	<0.001	0.70
ADFI, g/d	1333.6^b^	1342.6^ab^	1383.5^a^	1378.9^a^	27.2	1324.5^b^	1397.1^a^	25.4	0.05	<0.001	0.78
G:F	0.594	0.594	0.593	0.582	0.016	0.586^b^	0.596^a^	0.015	0.22	0.05	0.98
Days 0 to 42											
ADG, g/d	430.9	440.0	453.6	444.5	10.4	444.5	440.0	8.6	0.27	0.65	0.54
ADFI, g/d	607.8	607.8	616.9	621.4	13.2	603.3	621.4	11.3	0.73	0.06	0.79
G:F	0.709^c^	0.725^ab^	0.734^a^	0.716^bc^	0.005	0.736^a^	0.706^b^	0.003	0.002	<0.001	0.04
Double	0.726^ab^	0.741^a^	0.738^a^	0.738^a^	0.007	–	–	–	–	–	–
Single	0.693^c^	0.709^bc^	0.730^a^	0.693^c^	–	–	–	–	–	–	–
Days 0 to 56											
ADG, g/d	517.1	526.2	539.8	530.7	10.0	521.6	807.4	8.6	0.13	0.13	0.55
ADFI, g/d	780.2	784.7	798.3	802.9	15.4	775.6^b^	807.4^a^	13.2	0.40	0.005	0.90
G:F	0.662^bc^	0.671^ab^	0.676^a^	0.661^c^	0.004	0.675^a^	0.660^b^	0.004	0.003	<0.001	0.12

^1^Medication: Control = no nursery medication; AVI21 = Avilamycin (Kavault, Elanco Animal Health, Indianapolis, IN) fed at 80.5 mg/kg for 21 d; AVI42 = Avilamycin (Kavault) fed at 80.5 mg/kg for 42 d; CAR21 = Carbadox (Mecadox, Phibro Animal Health, Teaneck, NJ) fed at 55.1 mg/kg for 21 d.

^2^SEM listed for significant medication program × stocking density interactions are pooled SEM.

^abc^Means within a row with different superscripts are different (*P* < 0.05).

From days 21 to 42, feed efficiency was highest in the AVI42 program, while CAR21 was the lowest, and AVI21 and control were intermediate (*P* < 0.05; Table 3). Feed efficiency improvements with avilamycin have previously been reported and may be due to reductions in disease pressure as it is believed that avilamycin prevents *E. coli* attachment in the intestine (Beckler et al., 2018; Greiner and Knopf, 2018). These results indicate enteric disease pressure was still prevalent beyond the first 21 d of the study as the program still receiving medication outperformed all other medication programs.

From days 42 to 56, pressure from stocking density was at its highest as pigs continued to grow, and medications were no longer included in any of the diet programs. During this time, there was a trend for ADG to be greatest in the AVI42 treatment pigs, followed by CAR21 and AVI21, then the controls (*P* = 0.06; Table 3). The same pattern was observed in ADFI from days 42 to 56 (*P* < 0.05; Table 3). These results indicate medication programs early in the nursery not only help pigs through enteric disease challenges but may also set pigs up for maintained increased performance after removal from the diets. In the case of this study, longer feeding of avilamycin proved to be most beneficial. These results agree with previous work that has shown avilamycin and carbadox both improve ADG when fed to pigs with *E. coli* challenges, with avilamycin fed over 42 d showing the greatest improvements (Greiner and Knopf, 2018; Thompson et. al., 2019).

An interaction between nursery medication program and stocking density was observed for G:F from days 0 to 42, at which time G:F was significantly lower in single-stocked pigs for all treatments other than AVI42, which was not different from the double-stocked pigs (*P* < 0.05; Table 3). Outside of this interaction, feed efficiency was highest in double-stocked pigs for all analyzed time points besides days 42 to 56 (*P* < 0.05; Table 3). These results are surprising as most previous work would indicate increased stocking density does not impact feed efficiency in the nursery phase (Wolter et al., 2002; Laskoski et al., 2021). One hypothesis is that single stocking reduces G:F because pigs have a chance to play with feeders more and potentially waste feed, or pigs overeat because there is less competition at the feeder. The cause of reduced feed efficiency in single stocked pigs observed in this study is unknown, and the impact of the AVI 42 treatment is believed to be a random anomaly.

For the overall trial period (days 0 to 56), AVI42 and AVI21 had the highest improvements in feed efficiency (*P* < 0.05; Table 3). Although there was no significant impact on ADG over the entire test period, pigs fed the AVI42 program had numerically the highest ADG, followed by CAR21, AVI21, and control treatments. In this study, AVI42 demonstrated the highest improvement in nursery pig performance in the face of a natural *E. coli* challenge.

### The Impact of Medication Program and Stocking Density on Morbidity and Mortality

Morbidity and mortality tended to be lower from days 0 to 21 in both avilamycin programs compared to carbadox and control programs (*P* = 0.06; Table 4). Looking at the overall periods from days 0 to 42 and days 0 to 56, morbidity and mortality did not significantly differ among medication programs. At each period measured, morbidity and mortality were lower in single-stocked pens than double-stocked pens (*P* < 0.05; Table 4). These results would indicate double stocked pens experienced an increased level of stress, from disease or otherwise, and regardless of stocking density, feeding avilamycin for the first 21 d improved morbidity and mortality.

**Table 4.** Effects of nursery medication program and stocking density on incidence of morbidity and mortality, %

	Medication program^1^				SEM	Stocking density		SEM	*P*-value		
Item	Control	AVI21	AVI42	CAR21		Double	Single		Med	Density	Med × Density
Days 0 to 21	1.2	0.8	0.8	2.1	0.47	1.7^a^	0.8^b^	0.40	0.06	0.02	0.86
Days 0 to 42	2.7	1.4	2.8	2.7	0.73	3.1^a^	1.7^b^	0.63	0.22	0.01	0.97
Days 0 to 56	3.0	1.6	3.0	3.2	0.55	3.6^a^	1.9^b^	0.41	0.15	0.003	0.94

^1^Medication: Control = no nursery medication; AVI21 = Avilamycin (Kavault, Elanco Animal Health, Indianapolis, IN) fed at 80.5 mg/kg for 21 d; AVI42 = Avilamycin (Kavault) fed at 80.5 mg/kg for 42 d; CAR21 = Carbadox (Mecadox, Phibro Animal Health, Teaneck, NJ) fed at 55.1 mg/kg for 21 d.

^ab^Means within a row with different superscripts are different (*P* < 0.05).

## Conclusion

In the first 21 d, the carbadox treatment and both avilamycin treatments improved feed efficiency and ADG compared to the control pigs, indicating both medications did help pigs overcome at least some of the challenges presented by *E. coli*. The feed efficiency and diarrhea incidence and severity improvements observed in the AVI42 treatment from days 21 to 42 indicate pigs in this study were still experiencing challenges beyond 21 d and continued support by medication was beneficial. It appears double stocking pigs did lead to an increase in challenge level, as measured by increased diarrhea incidence and scores throughout the study, but with longer feeding of avilamycin, pigs were able to overcome the increased challenge and perform similarly to their single-stocked counterparts. Looking at the overall period, this study demonstrated similar growth performance but superior feed conversion in nursery pigs fed avilamycin compared to carbadox, regardless of length of feeding. Only the AVI42 treatment significantly impacted the incidence and severity of diarrhea compared to control pigs for the overall study period, indicating that longer feeding duration was needed to have an impact on diarrhea score and incidence once medication was removed from the diet. This study indicates avilamycin may be a suitable replacement for carbadox in a natural *E. coli* challenge when fed for 21 d, with potential for additional improvements with longer feeding dependent on disease status and duration of the challenge.

## Abbreviation

E. coli: *Escherichia coli*
